# Supplementary material for: The Safety Profile of General and Local Anaesthetic Agents: Data Collected during 20 Years of Spontaneous Reporting Activities in the Campania Region (Southern Italy)
Source: Pharmaceuticals (Basel). 2021 Dec 3;14(12):1261. doi: 10.3390/ph14121261 (PMC8703922; doi:10.3390/ph14121261)
Supplement: Supplementary file 1 [file pharmaceuticals-14-01261-s001.zip › pharmaceuticals-1448723-supplementary.pdf]

Supplementary Table S1. Italian macro-regions, regions and number of inhabitants, including the study's site (Campania Region in bold)

Italian macro-regions	Italian regions	Inhabitants (Update to January 2021)
Northern Italy	Lombardia	9.966.992
Central Italy	Lazio	5.720.796
**Southern Italy**	**Campania**	**5.679.759**
Northern Italy	Veneto	4.852.453
Italian islands	Sicilia	4.840.876
Northern Italy	Emilia-Romagna	4.445.549
Northern Italy	Piemonte	4.273.210
Southern Italy	Puglia	3.926.931
Central Italy	Toscana	3.668.333
Southern Italy	Calabria	1.877.728
Italian islands	Sardegna	1.598.225
Northern Italy	Liguria	1.509.805
Central Italy	Marche	1.501.406
Southern Italy	Abruzzo	1.285.256
Northern Italy	Friuli-Venezia Giulia	1.198.753
Northern Italy	Trentino Alto Adige	1.078.460
Central Italy	Umbria	865.013
Southern Italy	Basilicata	547.579
Southern Italy	Molise	296.547
Northern Italy	Valle d'Aosta	123.895

Source: dati.istat.it
